# Medical Societies and Organizations

**Published:** 1876-05

**Authors:** 


"We are aware that you, as the editor of a legitimate medical journal, favor medical societies and organizations which are established in strict accordance with medical ethics and the time-honored principles which governed these societies in our earlier professional days; but of late I have despaired of ever again seeing a society established on a high and liberal basis. Rings and cliques control alike both medical and political organizations, and appointments of honor are conferred in conformity with the narrow and selfish views of the *few* that control them. For these reasons it is impossible for societies to be kept up in many sections of our country, as many of our older and most talented men decline to attend these organizations."

What our correspondent says in the above extract is, we must confess, true, and lamentably so in regard to the conduct of some of these societies.

It applies to all sections alike, and though worse now than formerly, it was ever true to some extent, and will doubtless be the case until the millennial dawn shall drive out the native selfishness and depravity of mankind. We trust then that our correspondent will not be discouraged, and that he will continue his valuable contributions to medical literature. For, like him, we are laboring, and have long labored without emolument, and so far as we know, without appreciation by the higher dignitaries who control these bodies. Perhaps it is, in a large degree, their own fault that many good men are thus passed by. They should not stand back, but go forward and co-operate with these bodies, and seek to direct them for good.

As to appointments of trust and honor, unless made upon merit, they usually fail to benefit the parties so honored, and recoil not less to their injury than to the injury of the cliques and rings who manage to have them conferred.

We have heard like complaints made of the American Medical Association, and the charge has been, in some instances, correct. Yet it does not follow that the presiding officer who makes these appointments is necessarily a bad man. He cannot know everybody, and being compelled to receive suggestions, is sometimes deceived as to the fitness or competency of his appointees.

We trust that no consideration of the kind referred to will cause any good and true members of the profession who can attend, on the 6th of July, at Philadelphia, to refuse doing so.

Such an opportunity of meeting with the talented and progressive men of the profession will not occur again, perhaps, until another Centennial shall have rolled around, when we shall all be in our graves. Contributions to surgery and medicine will go up from all sections of the land, and foreign nations will vie with us in the exhibition of everything illustrative of the development and progress of medical knowledge, of art, and of every department of science. It is conceded that Philadelphia stands at the head of the list on this continent in her devotion to medical science. The senior editor of this journal is proud to claim Philadelphia as the place of his Alma Mater, and proud to know that the colleges of that city stand pre-eminently high, and that her teachers and representative men are not surpassed even by the best talent that can be found in the old country.

